# Primary and Secondary Abscission in *Pisum sativum* and *Euphorbia pulcherrima*—How Do They Compare and How Do They Differ?

**DOI:** 10.3389/fpls.2015.01204

**Published:** 2016-01-26

**Authors:** Anne K. Hvoslef-Eide, Cristel M. Munster, Cecilie A. Mathiesen, Kwadwo O. Ayeh, Tone I. Melby, Paoly Rasolomanana, YeonKyeong Lee

**Affiliations:** Department of Plant Sciences, Norwegian University of Life SciencesAas, Norway

**Keywords:** induced abscission, primary abscission, secondary abscission, pea, poinsettia, *def* mutants, Differentially expressed genes, RNA *in situ* hybridization

## Abstract

Abscission is a highly regulated and coordinated developmental process in plants. It is important to understand the processes leading up to the event, in order to better control abscission in crop plants. This has the potential to reduce yield losses in the field and increase the ornamental value of flowers and potted plants. A reliable method of abscission induction in poinsettia (*Euphorbia pulcherrima*) flowers has been established to study the process in a comprehensive manner. By correctly decapitating buds of the third order, abscission can be induced in 1 week. AFLP differential display (DD) was used to search for genes regulating abscission. Through validation using qRT-PCR, more information of the genes involved during induced secondary abscission have been obtained. A study using two pea (*Pisum sativum)* mutants in the *def* (*Developmental funiculus*) gene, which was compared with wild type peas (tall and dwarf in both cases) was performed. The *def* mutant results in a deformed, abscission-less zone instead of normal primary abscission at the funiculus. RNA *in situ* hybridization studies using gene sequences from the poinsettia differential display, resulted in six genes differentially expressed for abscission specific genes in both poinsettia and pea. Two of these genes are associated with gene up- or down-regulation during the first 2 days after decapitation in poinsettia. Present and previous results in poinsettia (biochemically and gene expressions), enables a more detailed division of the secondary abscission phases in poinsettia than what has previously been described from primary abscission in *Arabidopsis*. This study compares the inducible secondary abscission in poinsettia and the non-abscising mutants/wild types in pea demonstrating primary abscission zones. The results may have wide implications on the understanding of abscission, since pea and poinsettia have been separated for 94–98 million years in evolution, hence any genes or processes in common are bound to be widespread in the plant kingdom.

## Introduction

Abscission is a beneficial process for plants themselves, since this is the mechanism for plants to discard unwanted or superfluous organs in a highly orchestrated manner. However, this developmental process cause seed shattering, fruit drop, flower abscission, and other loss of value for crops valuable to man. It is not surprising that prevention of seed shattering probably was one of the first characters selected for when man started to cultivate plants and selected for cereal plants where he could harvest more seeds (Harlan et al., 1973). Abscission is a complicated process, it is not clear the orchestrated manner by which abscission is controlled in plants. The process is important to understand, since agricultural and horticultural production is increasingly more sophisticated and facilitates precise control of the growth conditions, in greenhouses and increasingly also in the field.

Cells in an abscission zone (AZ) are typically small, square-shaped with dense cytoplasm (Sexton and Roberts, 1982) and clearly distinguishable from surrounding cells. The number of cell layers in an AZ is fixed for a species, but is highly variable between species, with tomato as an example of two discrete cell layers, which split between them (Valdovinos and Jensen, 1968; Tabuchi et al., 2001). The AZ of *Sambucus nigra* on the other hand, is composed of up to 50 cell layers (Taylor and Whitelaw, 2001). The term secondary abscission zones was first introduced by Lloyd (1913-14). He reported on injury-induced abscission in *Impatiens sultani*. Secondary abscission has also later been described as a zone which occurs in a position where a zone would not normally form in an intact plant (Webster, 1970; Pierik, 1973). Having termed these adventitious AZ as secondary, the predestined AZ occurring at particular sites of positional differentiated cells have since been given the term primary (Huang and Lloyd, 1999) to distinguish between the two.

Abscission can be affected by environmental factors and is a highly coordinated biological mechanism (Brown and Addicott, 1950; Osborne, 1955; Addicott, 1982; Patterson, 2001; Roberts et al., 2002). It has been reported that low light conditions might trigger cyathia abscission in poinsettia (*Euphorbia pulcherrima*) (Bailey and Miller, 1991; Moe et al., 1992) but environmental regulations, as well as the biological background of abscission has not been fully investigated. Although the abscission process is a natural biological process to dispose of redundant organs, premature abscission results in the loss of yield and value in agriculture and horticulture.

Valdovinos and Jensen demonstrated the cell wall disintegration in the AZ allowing separation in tomato and tobacco (Valdovinos and Jensen, 1968). Reviews have followed with more insight into the process (Sexton and Roberts, 1982; Osborne and Morgan, 1989; Taylor and Whitelaw, 2001; Bosca et al., 2006). Our own results have clearly demonstrated and confirmed that abscission is controlled by inter-organ signaling events, yet it is still not clear how these signals co-ordinate the events. Cell wall modifications in the AZ of poinsettia, visualized using antibodies during the course of an induced abscission process, is one way we have chosen to elucidate upon the abscission process (Lee et al., 2008). Some of the other approached will become clear in the present article.

Poinsettia is not the obvious choice for fundamental studies since the molecular tools available for other model plants are not available. Secondly, the life span is much longer than for *Arabidopsis*. Thirdly, it is vegetatively propagated, does not readily set seed and segregation studies would be difficult to perform. Lastly, it has no available non-abscising mutants. However, poinsettia is an important ornamental plant worldwide during Christmas time. It is by far the most important potted plant crop in Norway with more than five million plants produced each year, for a population of about the same number. In addition, Norwegian poinsettia growers have pointed out that poinsettia suffers from premature flower abscission, which can result in severe losses in value. Therefore, there are economic reasons for being able to control this. A method for induction of abscission to investigate the abscission process has been developed using this plant species (Munster, 2006). This makes the study of abscission in poinsettia very precise and predictable. Poinsettia flower pedicels have no pre-destined AZ, and hence they are defined as having secondary abscission. This inducible abscission system in the poinsettia flower resembles systems in other plant species (Webster, 1970; Hashim et al., 1980; Oberholster et al., 1991; Kuang et al., 1992), especially the model plant tomato (pedicel abscission) and thus provides a reliable, synchronized system for studying the abscission process in general. Poinsettia (*E. pulcherrima*) belongs to the large family of *Euphorbiaceae*, with about 300 genera and 7500 species. A number of plants of this family are of considerable economic importance. Prominent plants include cassava (*Manihot esculenta*), physic nut or Barbados nut (*Jatropha curcas)*, castor oil plant (*Ricinus communis)*, and the Para rubber tree (*Hevea brasiliensis)*. Amongst several of these, genomic and molecular tools are becoming available, because of their economic importance for producing biofuels.

Previously, this inducible system has been used to study the turnover of carbohydrates in the abscission zone (Lee et al., 2008), and the effect of the cut position on hormones in the bud (Munster, 2006). This article reports on the genes differentially displayed during the 7 day period from induction to abscission from the thesis of Munster (2006). We have since further verified the gene expressions in poinsettia through quantitative RT-PCR and RNA *in situ* hybridizations, all of which is included in this article.

Poinsettia has no mutants for abscission, in order to study gene expression. However, there are other model systems with numerous mutants. There are two *def* (*Developmental funiculus*) mutant peas in the John Innes Pea Collection, one dwarf and one tall type (JI184 and JI3020). These mutants were the tools to elucidate upon the process of abscission in peas (Ayeh, 2008). That study concluded that the *def* gene is a single locus gene (Ayeh et al., 2011) and the abscission zone between the funiculus and the pea was characterized (Ayeh et al., 2009) in both mutants (with abscission-less zones) and tall and dwarf wild types. Pea has primary abscission, with the AZ clearly defined from the onset in the wild types and only the distorted abscission-less zone in the mutants.

Pea and poinsettia are separated by 94–98 million years (Bennett et al., 2000). Hence, any genes they share during the abscission process will most likely be universal throughout the plant kingdom. This paper summarizes the interesting comparable results in pea and poinsettia with respect to cell wall alterations (Ayeh, 2008; Lee et al., 2008) as well as gene expression during the abscission process from induction to abscission. Our hypothesis is that the primary abscission in pea (*Pisum sativum)* and secondary abscission in poinsettia (*E. pulcherrima)* are more similar than different. Pea represents primary abscission where the abscission zones are clearly defined from the development of the organs. Poinsettia represents secondary abscission, where the abscission zones can develop upon induction. This paper presents results, which tests the hypothesis comparing the developmental stages in poinsettia abscission with the *def* mutants and wild types in pea, discussing the similarities and differences between these two systems as models for abscission.

## Materials and methods

### Plant material—poinsettia

Poinsettia (*Euphorbia pulchérrima*) ‘Lilo’ were grown as previously described in Lee et al. (2008). Plants were grown under long day condition (20 h photoperiod at 150 μmolm^−2^sec^−1^) and the plants were kept under short day conditions (10/14 h photoperiod) at 20°C to induce flowering.

#### Induction of abscission in the flower pedicel (secondary abscission)

**Figure 1:**
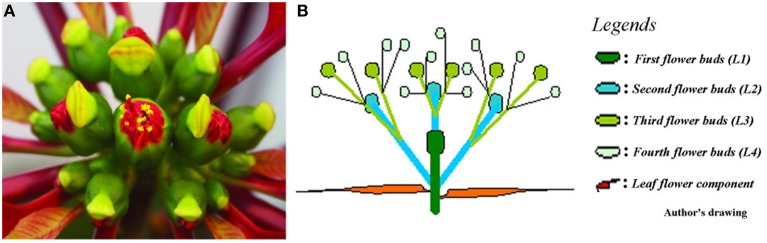
**Poinsettia inflorescence**. **(A)** Photo of a fully developed poinsettia inflorescence in aerial view. **(B)** Schematic drawing of the inflorescence showing 1st, 2nd, 3rd, and 4th order flowers in a profile view.

**Figure 2:**
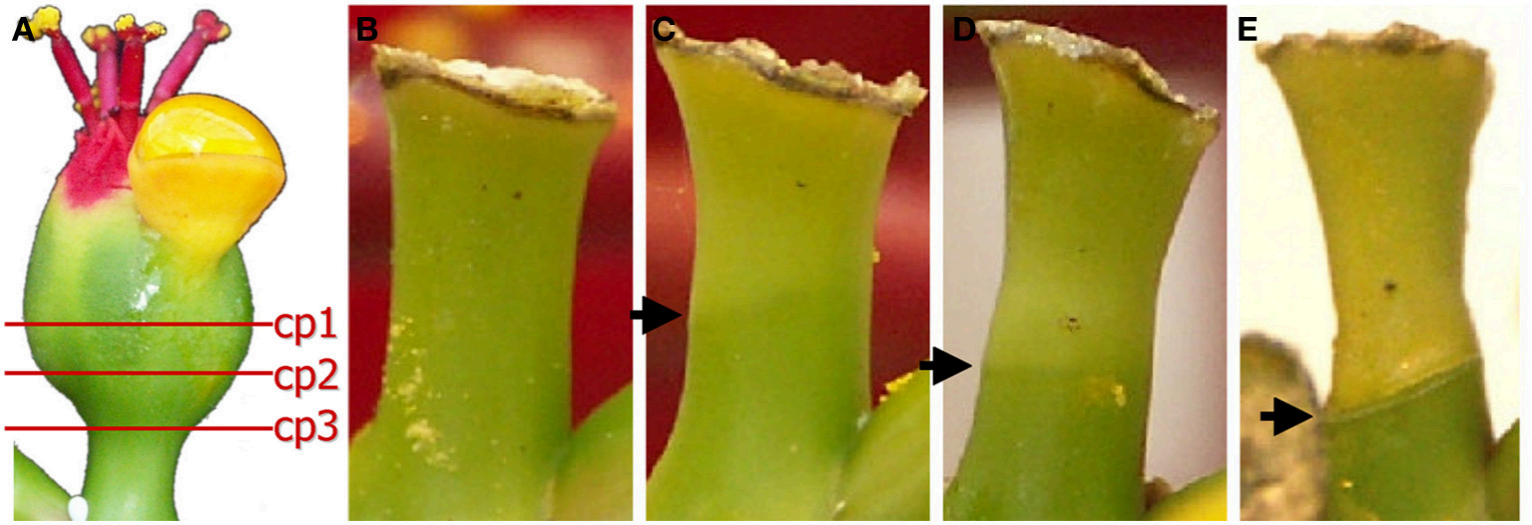
**Different cut positions (cp) (A) and development of the abscission zone (B–E)**. **(A)** The positions of the decapitating poinsettia cyathia (flowers) to induce controlled abscission. We have used cp2 in all experiments reported here. **(B)** Day 0 (control). **(C)** Day 5. **(D)** Day 6. **(E)** Day 7. Arrows indicate the AZs on the flower pedicels.

**Figure 3:**
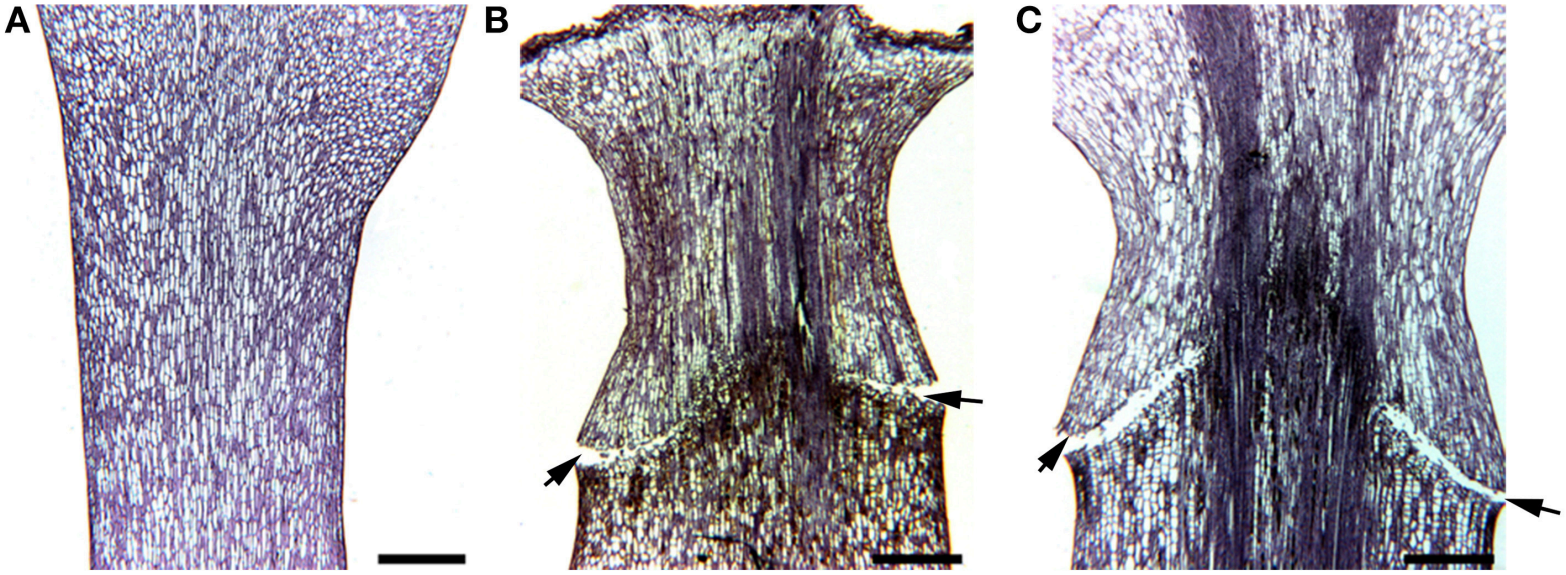
**Micrographs of poinsettia pedicels**. **(A)** A control without abscission. **(B)** An AZ induced by decapitation of flower bud at the right cut position. **(C)** A naturally formed AZ. Scale bars are 400 μm. Arrows indicate the AZs on the flower pedicels.

Cyathia of the third order (all male flowers) were used for analyses to standardize abscission zone development, since this gives six flowers in the same inflorescence of the same developmental stage (3rd order; Figure 1). When third order flowers began to open, they were decapitated with a razor blade just below the floral organs, with the floral bottom still intact, cut position 2 (cp2) in Figure 2A (Munster, 2006). The flowers developed abscission zones (AZs) under short day conditions, with 7 ± 1 days to complete abscission. AZs were dissected from the decapitated pedicels every 24 h, and harvested on the same time to create the complete series from Day 0 (control) to Day 7 and obtain comparable gene expressions. Figures 2B–E shows the development of the AZ from Day 0 to Day 7 (day of abscission). Figure 3 shows micrographs of poinsettia comparing a pedicel with no AZ (A) with induced (B), and natural (C) abscission.

### Plant material—pea

The four lines of pea (*P. sativum* L.) seeds (JI 116, JI 2822, JI 1184, and JI 3020) in this study were selected based on the presence of specific alleles at the *Def* locus, which control the detachment of the seed from the funiculus (Ayeh et al., 2009, 2011). Two wild types (WT) with the *Def* locus and two *def* mutant pea seeds were kindly supplied from the John Innes Pisum Collection Ayeh et al. (2009). Tall wild type (JI 116) and dwarf wild type (JI 2822) develop normal abscission events and therefore abscise the seed from the funiculus through the intervening hilum region. The tall *def* mutant (JI 1184) and the dwarf *def* mutant (JI 3020) both lack the abscission event and therefore fail to abscise the seed from the funiculus. These lines have a deficient abscission zone, which we have given the name abscission-zone-less (AZL; Ayeh, 2008). Seeds of each line were sown in pots with fertilized peat and grown under greenhouse conditions at 22°C and 16/8 h photoperiod with a photon flux of 110 μmol m^−2^ s^−1^ [400–700 nm Photosynthetic Active Radiation (PAR)] and a daylength extending light provided from incandescent lamps (OSRAM, Germany).

#### Definition of growth stages in pea

We used young and mature developmental stages in both the wild and the *def* mutant pea plants. For the tall wild type JI 116, developmental stage 10.1 indicates young seed. For the tall *def* mutant type JI 1184, developmental stage 8.1 indicates young seed for a comparable developmental stage. The developmental stage 2.1 indicates mature seed for both JI 116 and JI 1184. For the dwarf wild type JI 2822 and the dwarf *def* mutant JI 3020, developmental stages 4.1 and 3.1 indicate young seeds, respectively. Developmental stage 1.1 indicates mature seed for both dwarf wild type JI 2822 and dwarf *def* mutant JI 3020.

### Differential display (DD) in poinsettia

AZs were dissected from the area of the pedicel from Day 0 until Day 7 as described in Munster (2006). As soon as the AZ could be defined visually, samples of the distal part were harvested as an internal control to eliminate senescence related genes from the bands picked from DD. The pedicel slices from AZ tissue were stored immediately in RNAlater (0.1 g/ml) at −20°C until use.

#### RNA extraction, differential display, and sequencing

RNA was extracted from the AZ tissues by a time course according to instruction of the Qiagen RNeasy Plant Kit. RNA was treated using RNase-Free DNase I Set (Qiagen). The quantity and quality of the RNA was measured by Nano Drop ND-1000 spectrophotometer (Nano Drop Technologies, USA).

Fluorescent DD was performed with the RNAspectra kit (GenHunter, Nashville, TN, USA) in duplicates as all reactions were performed with both red (rhodamin) and green (fluoricin) fluorescence in parallel. The mRNA in the total RNA samples was converted into DNA by reverse transcriptase with anchor primers (H-T11A, H-T11G, or H-T11C). The resulting cDNA was template and PCR products were amplified by DNA Polymerase DyNAzymeII (Finnzyme, Espoo, Finland) using the three different anchor primers, respectively, for each arbitrary primer in separate reactions (for details see Supplementary Table 1). The RNA spectral kits used were red and green no. 1, 4, 5, and 8. Four primers were in addition designed on conserved areas of polygalactorunase and β-1,3-glucanase and used in the AFLP DD analyses. These primers were added since these two enzymes are associated with cell wall breakdowns during abscission. These primers were PG-A 5′-AAGCTTATTATGGAGC-3′, PG-T 5′-AAGCTTATTTTGGAGC-3′, GlucC 5′-AAGCT TTATGGAATG-3′, and GlucA 5′- AAGCTTTATGGCATG -3′. The amplification products were separated on 6% denaturing polyacrylamide gels casted between low fluorescence glass plates (Amersham Bioscience). Parallel amplification products (fluoricin or rhodamin) were separated on different gels and scanned on Typhoon 8600 (Amersham Bioscience, UK) using the following laser settings: flouricin; excitation 495 nm, emission 520 nm, green laser (532 nm), emission filter 526 SP and rhodamin; excitation 570 nm, emission 590 nm, green laser (532 nm), emission filter 580 BP 30. Kapton tape (Amersham Bioscience, UK) was used for gel orientation. The digital gel image was printed on paper size 1:1 and used for gel orientation and band identification. The AFLP gels were scored visually in duplicates, and the differentially expressed bands excised from the gel. DNA from the fragments was eluted in distilled water, precipitated and reamplified by PCR as described by the fluorescent DD kit manufacturer (GenHunter, USA). The PCR product was purified on an agarose gel. Single bands larger than 200 bp were excised and subcloned into plasmid pCR 2.1-TOPO (Thermo Fisher Scientific, USA) and chemically transferred into Top10 *Escherichia coli* cells as described by the manufacturer. Twelve positive *E. coli* colonies were selected, restreaked, and analyzed by colony PCR. The inserts were confirmed by separating the PCR products on an agarose gel. Plasmids from eight *E. coli* clones were prepared using Montage plasmid miniprep 96 (MERK Millipore, Germany) and Jetquick Plasmid Purification Spin Kit (Genomed, Germany). The insert was sequenced with BigDye Terminator Cycle Sequencing Kit v3.1 and ABIprism 3100 (MERK Millipore, USA). Sequences were visualized and processed in BioEdit sequence alignment editor (Hall, 1999).

#### Bioinformatics and putative homology identification of sequences

To identify homologous sequences of those differentially expressed during induced secondary pedicel abscission in poinsettia, different Blast [BlastN 2.2.30+, Database GenBank no (All GenBank + EMBL + DDBJ + PDB sequences) and standard settings] methods were used (Altschul et al., 1990). We also used Blast2Go to examine the ontology. All the Blast searches were repeated on August 30 2015. Most commonly used was Discontinuous MegaBlast (Morgulis et al., 2008), BlastN, and BlastX 2.2.32+.

### Real-time qRT-PCR for verification and quantification

Real-Time qRT-PCR primers were constructed using Primer Express (Thermo Fisher Scientific, USA; Supplementary Table 1). The primers were tested on both cDNA and genomic DNA. Real time qRT-PCT analyses for the short sequences were performed with a 7900HT Fast Real-Time PCR System (Thermo Fisher Scientific,) using SuperScript III Platinum Two-Step qRT-PCR Kit (Thermo Fisher Scientific). Transcript levels were normalized using poinsettia 18S primer pair to make correlative gene expression measurements (Table 1). All reactions were done in triplicate using two different biological preparations.

**Table 1:** **Real-time RT-PCR results and similarity searches of AFLP DD clones from poinsettia pedicel secondary abscission**.

**DD#**	**Primers used**	**GenBank Acc. #**	**Size (bp)**	**Real time RT-PCR results^a^**	**Discontinuous MegaBlast, BlastN, or BlastX**				
				**Days after abscission induction**					
				**0 1 2 3 4 5 6 7 Dist^e^**	**Accession no**.	**Description**	**Species**	**Id (of 100%)**	***E*-value**
**025a**	29A	EB647682	194	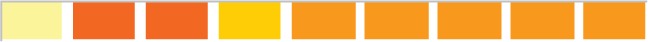	GQ856147	Unknown mito. genome region	*Citrullus lanatus*	91^b^	9e-50
**006a**	33G	EB647681	269	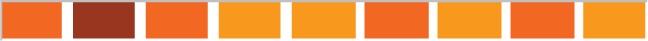	XP_007012396	Photosystem II subunit X	*Theobroma cacao*	86^d^	3e-12
					AY340642	UVB-repressible protein	*Trifolium pratense*	79^b^	5e-31^**^
**220**_	29C	EB647701	515	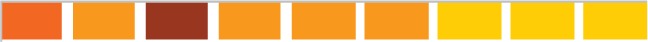	XM_002528833	Transcription factor	Ricinus communis^*^	81^b^	5e-26
**084**_	40A	EB647705	456	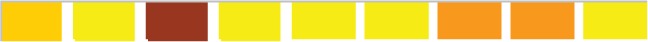	AEJ07931	Opie3 pol protein	*Zea mays*	41^d^	4e-24
**090a**	26A	EB647690	255	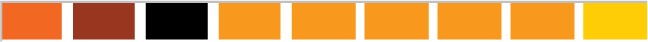	XM_011094475	Organ-specific protein	*Sesamum indicum*	71^c^	0.020
					AY188755	Atypical receptor-like kinase	*Zea mays*	86^c^	8.2^**^
**038b**	62C	EB647683	233	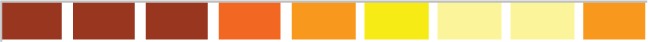	CP011890	Unknown region of chr. 5	*Ovis canadensis*	86^c^	0.005
304b	PGTC	EB647707	217	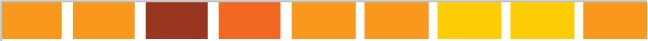	HQ874649	Unknown mito. genome region	Ricinus communis^*^	96^b^	1e-61
057b	34A	EB647687	412	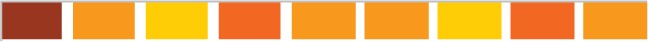	YP_002720125	Cytochrome f subunit (PetA)	Jatropha curcas^*^	96^d^	1e-65
208_	27A	EB647706	443	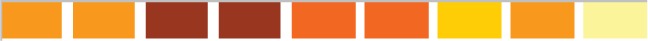	AY794600	Chloroplast tRNA-Leu	E. pulcherrima^*^	99^b^	3e-98
136b	GlcG	EB647697	447	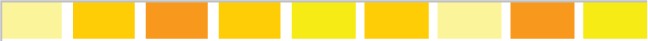	XM_002512633	fk506-binding protein	Ricinus communis^*^	81^b^	3e-47
045c	1C	EB647684	347	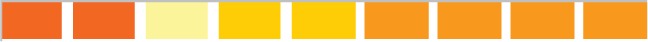	XM_002510884	Uridylate kinase	Ricinus communis^*^	83^b^	2e-35
140b	PG0	EB647699	306	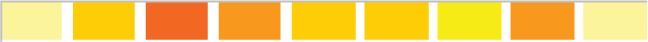	XM_002532624	eIF3E (translation initiation)	Ricinus communis^*^	84^b^	2e-28
103_	GlaG	EB647693	434	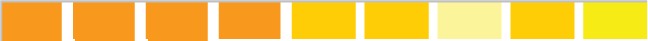	XM_012213718	CASP-like protein	Jatropha curcas^*^	77^b^	8e-36
003a	33A	EB647680	279	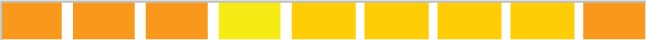	FJ228477	α-tubulin	*Betula pendula*	92^b^	2e-21
301_	PGA	EB647702	448	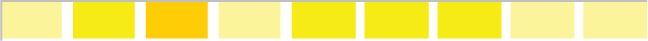	XM_002512412	RNA binding protein	Ricinus communis^*^	74^b^	1e-20
060c	34A	EB647688	304	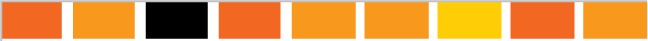	XM_010526012	V-ATPase G subunit 1	*Tarenaya hassleriana*	83^b^	2e-09
320b	PGTG	EB647704	218	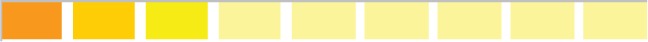	BT092277	unknown mRNA	*Glycine max*	87^b^	6e-07
082a	40G	EB647689	269	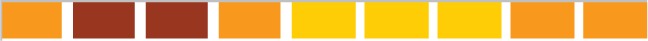	XM_002523025	Putative β-glucosidase	Ricinus communis^*^	72^c^	1e-06
101_	GlaG	EB647692	426	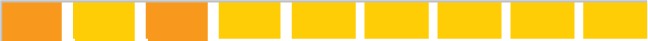	XM_002511291	Histone deacetylase	Ricinus communis^*^	71^c^	6e-06
140a	PG0	EB647698	307	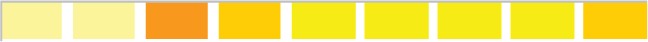	AM932356	Partial tRNA-Leu gene	*Typhonium giganteum*	73^c^	1e-05
091b	26G	EB647691	247	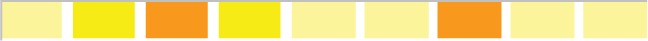	CP001685	Glucan endo-1,3-β -D-glucosidase	*Leptotrichia buccalis*	90^c^	0.019
047b	6C	EB647685	236	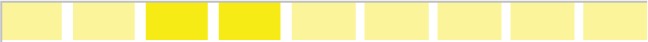	XM_002305663	Proteasome beta subunit B family protein	*Populus trichocarpa*	82^b^	7e-08
130_	GlcG	EB647696	297	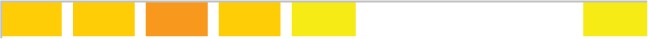	AY792209	NADH dehydrogenase SU 4L	*Ceratitis neostictica*	81^c^	3.5
304a	8C	EB647686	299	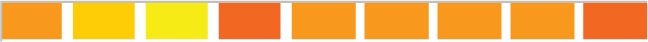	–	No significant matches	–	–	–
304a	GlaG	EB647694	343	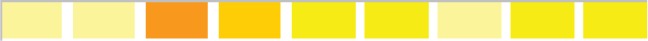	–	No significant matches	–	–	–
304a	GlcG	EB647695	268	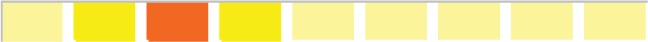	-	No significant matches	–	–	–
304a	GlcG	EB673117	249	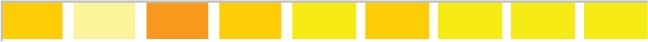	-	No significant matches	–	–	–
204c	27G	EB647700	214	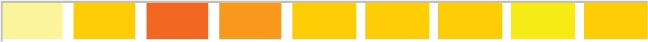	XM_012216503	Proteasome subunit alpha type-1-B-like	Jatropha curcas^*^	72^c^	1e-10
304a	PGA	EB647703	463	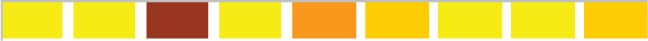	–	No significant matches	–	–	–

*Similarities found in DNA sequences from chromosome, mitochondria, chloroplasts or bacteria. List of primers in Supplement Materials*.

a *Visualization of Real-time RT-PCR results relative to 18S expression of two individual experiments. The highest expression is black and the lowest expression is white, showing decreasing nuances of orange for descending steps of equal size (8 nuances/steps)*.

b *Discontinuous MegaBlast [BlastN 2.2.32+, Database GenBank no (All GenBank + EMBL + DDBJ + PDB sequences) and standard settings 30 August 2015*.

c *BlastN [BlastN 2.2.32+, Database GenBank no (All GenBank + EMBL + DDBJ + PDB sequences) and standard settings 30 August 2015*.

d *BlastX Database no and standard settings 30. August 2015. Reading frame (RF) +1 of the Acc. # EB647681, +3 of the Acc. # EB647705 and EB647687*.

e *Internal control: Distal part of the abscising pedicel above the abscission zone at Day 0*.

* *Euphorbiaceae family member*.

** *From BlastX in Sept 2012, incl because they give an additional indication of function*.

qRT-PCR reactions for the whole gene sequences were performed with a 7700 Real time PCR system (MERK Millipore) used Platinum®SYBR®Green qPCR SuperMIX-UDG with ROX according to the manual (Thermo Fisher Scientific). The qRT-PCR was carried out in 25 μl reactions using 2.5 μl of diluted template, 0.5 μl of each primer (stock10 μM- final 0.2 μM) and 1x SYBR Green reaction mix. Template, cDNA, were diluted 10^−1^ and 10^−4^ for the reactions included RACE-primers and 18s primers, respectively. Triplicate repeats of each reaction and a template control of nuclease free water was carried out. Amplifications were performed with the following program: 95°C for 2 min followed by cycles of 95°C for 15 s and 60°C, 30 s. After amplification a melting curve analysis was performed. An internal reference dye, ROX, was included in the Platinum SYBR Green buffer to normalize the fluorescent reporter signal in real-time quantitative RT-PCR.

### Whole gene sequencing by 5′rapid amplification of cDNA ends (5′race)

The total RNA from the AZ-tissue in poinsettia was used as template to synthesize first strand cDNA in a reverse transcription reaction using modified oligo (dT) primer. Gene-specific primers (GSP) were constructed from seven of the DD-sequences, using Primer 3 Software (http://frodo.wi.mit.edu). For the GSP to find the correct cDNA-sequences the RACE reaction was optimized to isolate the complete gene sequence. The seven sequences were picked on the basis of showing interesting DD differences, but too short for qRT-PCR and Blast searches initially. The primers used are shown in Table 2.

**Table 2:** **AFLP DD and RACE primers, results and similarity searches for full-length genes in poinsettia secondary abscission**.

**DD#**	**Primers used for DD**	**Primers used for 5′ RACE**	**AFLP DD results^a^**								**BlastX^b^**				
			**Days after abscission induction**												
			**0**	**1**	**2**	**3**	**4**	**5**	**6**	**7**	**Accession no**.	**Description**	**Species**	**Id (of 100%)**	***E*****-value**
135		5′gactttccgtcccccatccctcatc 3′	+	–	–	–	–	–	–	–	XP_002518733	Polyadenylate-binding protein 2	Ricinus communis^*^	86	8e^−155^
133a		5′cagagtgccatgtcacctcgaacct 3′	–	+	–	–	–	–	–	–	NP_176471	Lys-specific histone demethylase 1-1	*A. thaliana*	73	8e^−138^
											Predicted	Lys-specific histone demethylase 1-1	*Prunus mume*	79	0.0
108		5′cccccaggcaacaaataagagtc 3′	+	–	–	–	–	–	–	–	XP_002530011	V-SNARE protein	Ricinus communis^*^	91	2e^−126^
122		5′catccccagtacgaatcccaatacg 3′	–	+	–	–	–	–	–	–	XP_012085745	Bidirectional sugar transporter N3	Jatropha curcas^*^	76	4e^−136^
82b		5′ggcaacaaccgcagaaagtcgtaac 3′	–	+	–	–	–	–	–	–	XP_002522811	Glycine-rich RNA-binding protein	Ricinus communis^*^	74	1e^−44^
105		5′ggccatgcaacatacaaccatc 3′	+	–	–	–	–	–	–	–	NP_198917	DNA-directed RNA Pol. II su. K	*A. thaliana*	88	6e^−24^
32a		5′gctctagctccatcaacccccaaag 3′				+	+	+		+	NP_001077782	DVL3	*A. thaliana*	58	1e^−5^

*Similarities found in DNA sequences from chromosomes*.

a *Reconstruction of visual screening of the result where + is upregulated and − is downregulated*.

b *BlastX (BLASTX 2.2.32+) Database no and standard settings 30 August 2015. Reading frame (RF) +2 of the Acc. # NP_198917, NP_001077782, XP_002522811 and XP_002518733. RF +1 of the Acc. # XP_012085745 and NP_176471. RF +3 of the Acc. # XP_002530011*.

* *Euphorbiaceae*.

The 5′-RACE was performed according to BD SMART™ RACE cDNA Amplification Kit (BD Biosciences Clontech, USA). The RACE products were characterized by cloning and sequencing. The 5′RACE products were cloned into the pCR®4-TOPO vector and transformed into competent TOP10 *E. coli* cells (Supplementary Table 2). The inserts were sequenced to verify that the amplified product had a segment of the same sequence as in the DD product and to obtain sequence information from the RACE product and its orientation in the 4-TOPO vector (Supplementary Table 3).

### RNA *in situ* hybridization of poinsettia and pea

Flower buds of poinsettia ‘Lilo’ induced for abscission and control plants were cut into small pieces (2–3 mm-thick) which were immediately fixed using 4% paraformaldehyde in sodium phosphate buffer pH 7.0 and 0.1% (v/v) Tween 20, under vacuum for 1 h, and left overnight at 4°C. After fixation, samples were washed in saline, dehydrated through a graded ethanol series, and embedded in paraplast (Sakura, Japan) using Tissue-Tek VIP Jr automatic embedding machine (Sakura, Japan). The 10 μm-thick sections were collected on poly-L-lysine coated slides.

Similarly, the primary abscission zones of the wild type peas (JI 116 and JI 2822), as well as the two *def* mutant peas (JI 1184 and JI 3020) were harvested and given the same fixation and embedding as the poinsettias above. The *def* gene is a single locus gene (Ayeh et al., 2011) and the abscission zone between the funiculus and the pea was characterized (Ayeh et al., 2009) in both mutants (with abscission-less zones) and both tall and dwarf wild types.

The 20 selected sequences for hybridization were reamplified from the pCR2.1TOPO constructs using their respective AFLP DD primers (Supplementary Table 4) and inserted into the pCR4 TOPO plasmid using the TA overhang cloning technology (Thermo Fisher Scientific, Germany). Single-stranded RNA probes were synthesized after linearization of plasmid DNA, using *Not*I and *Spe*I restriction enzymes for sense and antisense probes, respectively. Sense and antisense probes labeled with digoxigenin (DIG). dUTP were prepared using T3 and T7 RNA polymerases (Roche, Germany), respectively.

The 10 μm-thick sections on poly-L-lysine coated slides were dewaxed using Histoclear (Cell Path, UK). The sections were treated with Proteinase K (1 μg ml^−1^) and acetylated using 0.5% acetic anhydrine (Sigma Aldrich, Switzerland) in 0.1 M triethanolamine and followed by washing in PBS solutions and dehydrations using a graded ethanol series. Per slide, 100-200 ng labeled antisense and sense riboprobes were applied in 40 μl hybridization solutions in a humid chamber for 16 h at 50°C. Hybridization was performed using hybridization solution containing 50% formamide, dextran sulfate, Denhardt's solution, tRNA, and 10 × hybridization buffer (3 M NaCl, 0.5 M Na_2_HPO_4_, 10 mM EDTA). After hybridization, sections were washed in SSPE and NTE buffer. After RNase treatment, sections were blocked and probe was detected using anti-digoxinenic-alkaline phosphate-coupled antibody. The hybridized DIG-labeled probe and target was detected by anti-DIG antibodies conjugated with alkaline phosphatase (Butler et al., 2001). Sections were visualized by applying 5-bromo-4-chloro-3-indolyl phosphate (BCIP) and nitroblue tetrazolium salt (NTB; Promega, USA) diluted in alkaline phosphate buffer. Sections were photographed using bright field optics (Leica, Germany).

## Results

### Genes differentially expressed during secondary abscission in poinsettia and their putative identity

To elucidate on secondary abscission related genes, AFLP DD analysis provided a database of poinsettia cDNA sequences involved in AZ development and the induced secondary abscission process. The total RNA samples used in the AFLP DD screening were prepared from AZ tissue at eight different stages: every 24 h from Day 0 (control) and until abscission on Day 7. After 3–4 days, the first visible signs of the forthcoming abscission event could be seen as a lighter green band at the place of the AZ and the onset of senescence related color changes of the distal end of the flowers (Figures 2B–E). On Day 7 the distal end normally abscise after a coordinated separation of the cell layers in the AZ. Generally, the induction of flower abscission in poinsettia by decapitation (Figure 2A) has an accuracy of ±1 day (data not shown).

**Figure 4:**
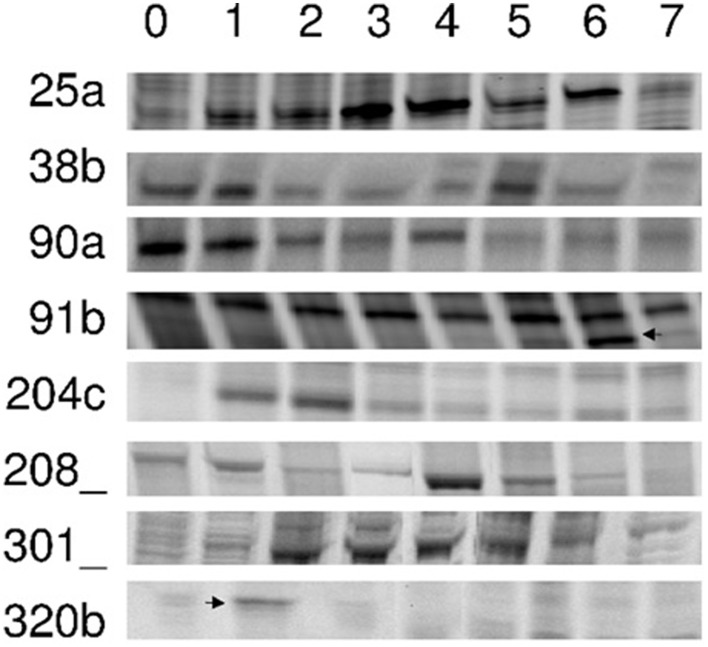
**DD-PAGE sections cDNA PCR products that were differentially expressed**. Poinsettia flower abscission zone total RNA samples were prepared from Day 0 to Day 7(0–7). Left column indicate specific clone numbers. Arrows are indicating specific bands isolated.

**Figure 5:**
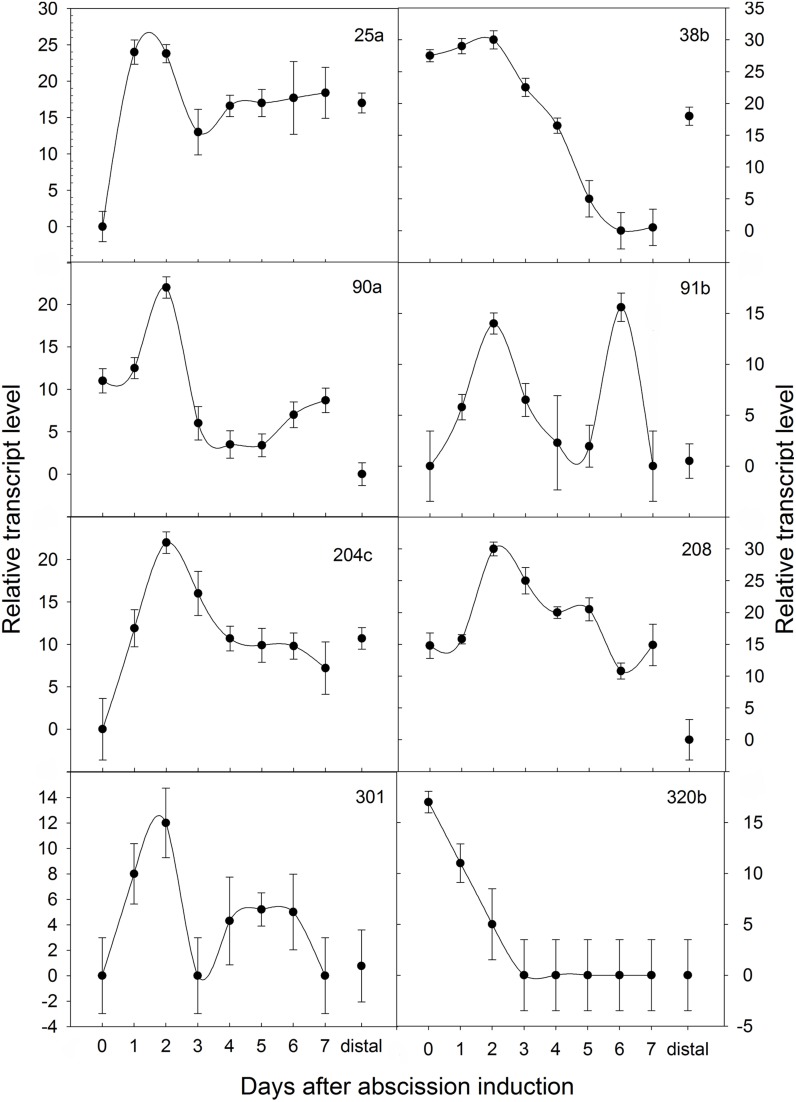
**Temporal expression patterns of poinsettia DD clones monitored by Real-Time qRT-PCR**. ΔΔCt on the y axis refers to the fold difference of a particular DD clone mRNA level relative to its lowest expression. Expressions were normalized to the 18S ribosomal RNA endogenous control during an induced abscission process of poinsettia pedicel, whereas distal is an internal control sample dissected from above the AZ.

Our screen gave us 126 gene transcripts, either up- or downregulated, an example gel from the DD is shown in Figure 4. The sequences which were >200 bp (74 sequences) were also run in a Real-Time quantitative assay (qRT-PCR), a selection of the most interesting are presented in Figure 5. Seven others, which were of particular interest due to their expression profiles in the DD, were then put through a 5′RACE and whole gene sequencing to include them in assay; making a total of 81 sequences tested. Almost half of the first 74 (35) were confirmed to be significantly differentially expressed relative to the ribosomal subunit 18 (18S) in the qRT-PCR assay. BLAST analysis gave a decent similarity hit for 29 sequences, where 18 matched beyond an expectation value of E^−4^ (Table 1). Eight of the sequences had no matches in databases, some of these were strongly up- or downregulated as early abscission events. They are putatively very interesting gene sequences to study further to find their specific functions, as potentially unknown AZ-genes. From the 5′-RACE-extended sequences we obtained matches beyond E^−5^ for all seven, some these also highly relevant (Table 2).

Blast “Top-hits” species distribution chart obtained by performing Blastx to NCBI, using Blast2go program, showed only two hits to *Theobroma cacao* and one hit to the following species: *Aegolops tauschi, Nicotiana sylvestris, Zea mays, R. communis, Datora stramonium and Medicago truncatula*. Of these species, *R. communis* belongs to the same family as poinsettia, namely *Euphorbiaceae*. Examining Table 1, *Japtropha curcas* also belongs to the same family. Although many exact hits in Tables 1, 3, Jatropha does not reach as high overall as the abovementioned species, and does not have more than one hit in Blastx.

**Table 3:** **Proposed division of abscission phases in poinsettia (0-IV), the corresponding # of days after decapitation and the percentage (%) of the 225 DD bands, which fall into each phase, either up- or downregulated**.

**Corresponding days after decapitation**	**0 (Control)**	**0–2**	**2–4**	**4/5–7**	**7**
Phases in abscission	0	I	II	III	IV
% sequences Upregulated	6	3	8	16	2
			4		
		19			
			11		
% sequences Downregulated	29	3			

*126 of these were successfully cloned and sequenced*.

Table 3 provides the percentage of up- and down-regulated genes belonging to each proposed phase in abscission of poinsettia. The majority of the significant differentially expressed genes were upregulated during the first or second day after the induction of abscission, in phase I. At this stage, it is not possible to see any changes in the anatomy of the pedicel or any color changes. Many of the genes can be functionally classified to energy metabolism, cell growth and division.

We have also identified two sequences similar to cell wall degrading enzymes (glucan endo-1,3-β-glucosidase and another putative β-glucosidase), some sequences, which can be associated with endoreduplication (fk506-binding protein and α-tubulin), as well as a putative signal transducer.

Our specific primers toward polygalactorunase and β-1,3-glucanase, showed differences in the AFLP screens, but could not be identified as such through the BLAST searches. We thus consider them as successful arbitrary primers that were all able to produce DD bands picked up for identification. Regarding the 32 arbitrary primer from GenHunter, large differences could be observed in their ability to give differentially displayed bands, only 10 out of 32 primers did. The 36 different arbitrary primers used would statistically give ~75% of all expressed cDNAs, based on calculations made by Yang and Liang (2004). In our hands, fluorescent probed primers were just as effective as radioactive labeled primers (data not shown), corresponding to that the two methods have comparable sensitivity (Ito et al., 1994). The rhodamin signal was generally slightly stronger than the fluoricin signal. Figure 4 shows eight examples of selected DD bands. From all DD bands, we obtained 126 sequences after cloning the reamplified DD bands into a pCR 2.1-TOPO vector. When sequenced, product lengths were between 180 and 520 bp (Munster, 2006). Some bands where reamplified as a shorter sequence than the one initially picked out from the AFLP DD gel, this can be explained by the fact that the primers used are arbitrary and the PCR conditions are changed from AFLP to reamplification.

An overview of all bands picked up in the AFLP DD (225 in total) shows that 29% (65/225) of the clones derived from DD bands are downregulated after Day 0, in Phase 0.3% (7/225) are down-regulated after Day 2, after Phase I. Approximately 6% (13/225, Phase 0) and 3% (7/225) of the bands were upregulated at Day 1 and 2, respectively, taken together about 9% of the DD bands were upregulated in Phase I. During Phase II (Days 2–4), 8% (18/225) of the DD bands were upregulated. On Days 3–5 (Phase II-III), 4% (10/225) of the DD bands were upregulated. During Phase III (Days 4/5–7), 16% (35/225) of the DD bands were upregulated. Two percent (5/225) of the DD bands were upregulated at Day 7, in Phase IV. In addition, 19% (43/225) and 11% (24/225) of the bands were seen upregulated at Days 1–7 (after Phase 0) and Days 2–7 (after Phase I), respectively. Of these bands, 126 were successfully cloned and sequenced.

For all sequences, similarity to known protein and DNA sequences were found using different Blast methods (Discontinuous MegaBlast, BlastN and BlastX) and standard settings (Tables 1, 2). Twenty-two of 29 sequences generated putative similarities, out of which 13 have a very high similarity (*E*-value < e^−21^), five have high similarity (*E*-value e^−10^–e^−4^), and four have a less high similarity (*E*-value e^−3^–8.2). The latter ones are also reported due to the fact that short sequences can show relevant hits with high *E*-value (Information, T.N.C.F.B., 2012). This can be illustrated by the similarity hit on Glucan endo-1,3-β -D-glucosidase in *Leptotrichia buccalis* by sequence 91b (Acc.# EB647691) with an *E*-value of 8e^−3^ (Table 1). Two of the sequences did not have a significant similarity, while two were annotated as unknown mRNA and one as unknown mitochondric region. In addition to the possibility of the two with no match, being a new and unknown sequence, a contributing factor might be the use of poly A selective primers, amplifying the non-translated UTR-3′ region.

The identified DD genes from Tables 1, 2 can be grouped according to the following biochemical functions: ***(1) Transcription*** [Transcription factor (XM_002528833) and Opie3 pol protein (RTV_2, AEJ07931) and DNA-directed RNA Pol. II su. K (NP_198917)]. ***(2) Signal transduction*** [Organ-specific protein/Atypical receptor-like kinase (XM_011094475/ AY188755), DVL3 (NP_001077782)] and ***(3) translation/protein synthesis*** (eIF3E (translation initiation (XM_002532624), Chloroplast tRNA-Leu (AY794600), RNA binding proteins (XM_002512412 and XP_002522811), Lys-specific histone demethylase 1-1 (NP_176471), and Polyadenylate-binding protein 2 (XP_002518733), Proteasome subunit alpha type-1-B-like (XM_012216503)]. ***(4) Energy*** (V-ATPase G subunit 1 (XM_010526012) and Bidirectional sugar transporter N3 (XP_012085745), energy metabolism (photosynthesis; Cytochrome f subunit (YP_002720125), respiration; NADH dehydrogenase SU 4L (AY792209), and unknown mitochondrial DNA regions (GQ856147 and HQ874649) and proteasome metabolism (Proteasome subunit β type-7-B-like (XM_002305663)]. ***(5) Cell growth and division*** [α-tubulin (FJ228477), V-SNARE (XP_002530011), and Histone deacetylase (XM_002511291)] and ***(6) Cell structure*** [cell wall degradation (Glucan endo-1,3-β -D-glucosidase (CP001685 and XM_0022523025)]. ***(7) Defense/Disease*** [Photosystem II subunit X/UVB-repressible protein (XP_007012396/AY340642) and an uncharacterized membrane protein (CASP-like protein, XM_012213718)]. Sequence 136b with the best similarity on the Cytochrome f subunit (1e^−47^) also has an alternative and interesting similarity in immunophilin (NP196845 (4e^−24^).

It is noteworthy that the length of the sequence did not seem to have an influence on the *E*-value (Table 1). Only a few sequences a little longer than 200 bp were not able to give any similarities to the GenBank databases using different available programs (August 2015).

#### RNA *in situ* hybridization of selected sequences

**Figure 6:**
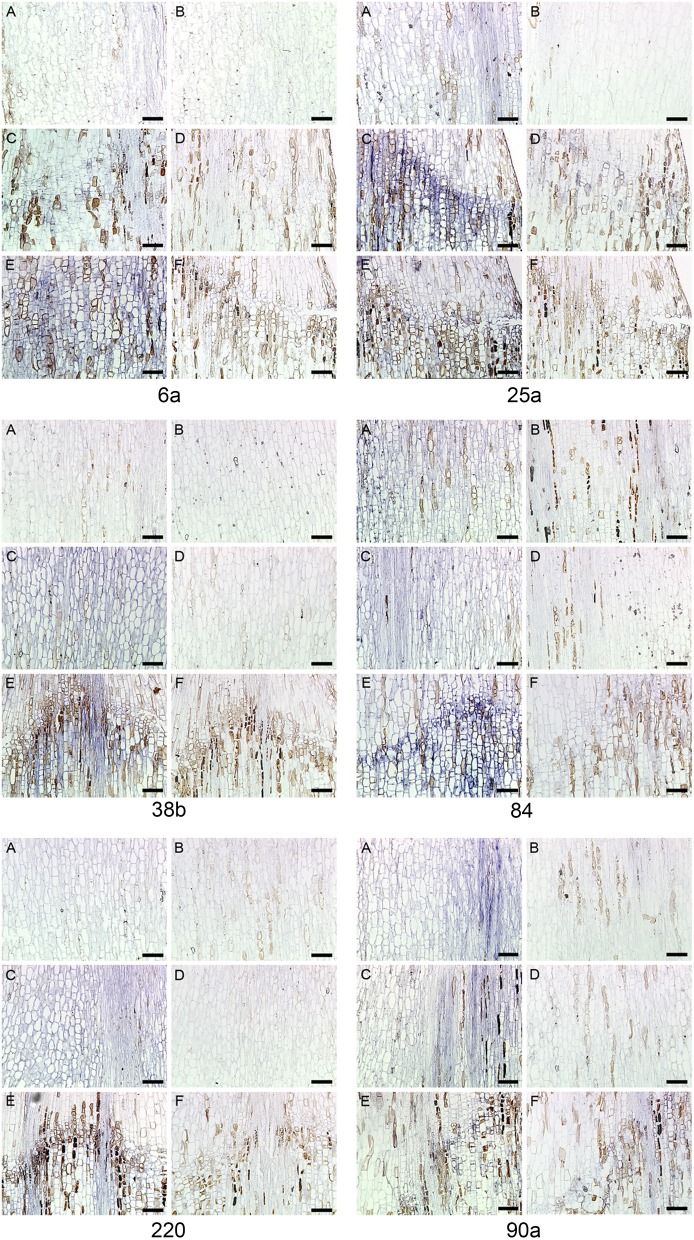
**RNA ***in situ*** hybridization six transcripts in ***Euphorbia pulcherrima*** (poinsettia) flower pedicels**. Control Day 0 **(A, B)**, Day 5 **(C, D)**, and Day 7 **(E, F)** after abscission was induced by decapitation. Longitudinal sections were hybridized with antisense **(A, C, and E)** or sense **(B, D, and F)**. The six DD riboprobes # *6a, 25a, 38b, 84, 220*, and *90a*. Bars: 100 μm.

Twenty of the DD sequences from Table 1 were also run through a RNA *in situ* hybridization to investigate further the spatial and temporal gene expression for these. The twenty were picked on the basis of which of the 29 gave good probes for RNA *in situ*. All the RNA *in situ* results verified them being expressed in the AZ and not elsewhere (data for six of them are shown in Figure 6). These six have been chosen based on the results from the other RNA *in situ* experiments, where these riboprobes were hybridized with sections of the four pea accessions from John Innes Centre, UK. These six riboprobes are on top of Table 1 and the DD # are in bold.

**Figure 7:**
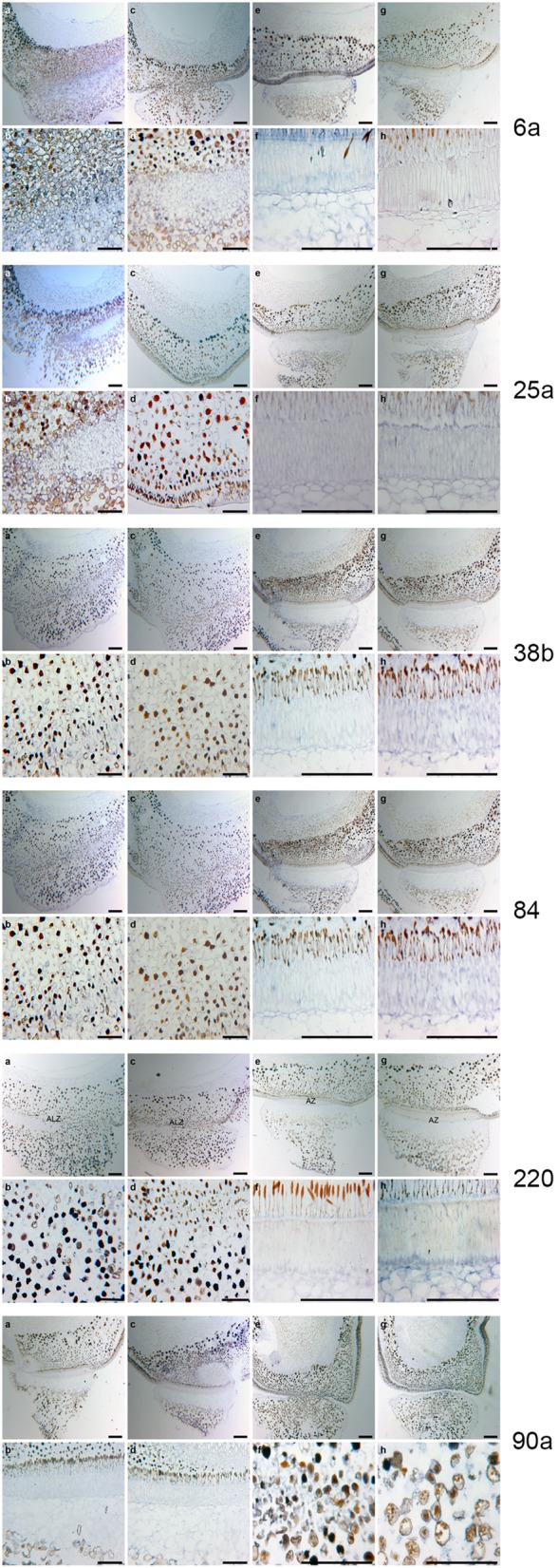
**RNA ***in situ*** hybridization for ***Pisum sativum*** using the six poinsettia riboprobes from the poinsettia DD. (A)** The antisense RNA localization of the *def* mutant JI 1184 for each of the six DD riboprobes # *6a, 25a, 38b, 84, 220*, and *90a*. **(B)** are the higher magnification of the As. **(C)** are the sense (control probe) of *def* JI 1184. **(D)** higher magnification of **(B)**. **(E)** antisense *Def* wild type JI 116. **(F)** higher magnification of **(E)**. **(G)** sense *Def* wild type JI 116. **(H)** higher magnification of **(G)**. Scale bars: **A, C, E**, and **G**, 12.5 μm; **B, D, F**, and **G**, 25 μm.

The six genes (6a, 25a, 38b, 84, 90a, and 220) are all differentially expressed both in poinsettia and in pea. The positive gene expression in antisense samples (Figures 6A,C,E, 7A,B,E,F) are seen as a dark blue coloring on the cells compared to no expression (light blue cells) in sense samples (Figures 6B,D,F, 7C,D,G,H). Ayeh (2008) showed that poinsettia riboprobes could be expressed in the AZ or the ALZ of the wild type and *def*-mutants (both the tall and the dwarf accessions). We found that five of these riboprobes were present only in the mutant peas (6a, 25a, 38b, 84 and 220), while only one was expressed in the wild type (90a).

Similarly, Rasolomanana (2008) was able to demonstrate through RNA *in situ* hybridization on sections of poinsettia pedicels that two of these six riboprobes (90a and 38b) are involved with the onset of abscission during the first 2 days. Another two (84 and 220) are associated with early-mid-term expression of the process. The last two (6a and 25a) were also expressed toward the end of the separation.

## Discussion

*E. pulcherrima* (poinsettia) can be used as a model system to elucidate on secondary abscission, induced by decapitation of the flower bud and studied through the 7-day period before abscission. When examining the natural abscission of a poinsettia bud, (Figure 3C) with that of a decapitated bud (Figure 3B), we can clearly see the similarities. The abscission process starts from the epidermis around the whole pedicel, and as the AZ develops toward the core of the pedicel, we see that the pedicel has continued to grow through cell division and cell elongations of the surrounding tissues, and the AZ is pushed upwards, forming a cone shaped stub as the bud falls off on Day 7. This cone shaped stub is very characteristic of poinsettia abscission. The control bud (Figure 3A) has not been decapitated and is the same age and from the same third order inflorescence as C. This bud has not reached the mature stage of the flower in the natural abscission (Figure 3B) yet, and shows no sign of developing AZ.

Although many plants have flowers and/or other plant parts that abscise, we know of few systems as accurate and foreseeable as this inducible poinsettia model system. Other model systems, as pea leaves (McManus et al., 1998), impatiens (Warren Wilson et al., 1986), and tomato (Kalaitzis et al., 1995), are all prepared explants *in vitro* and not *in planta*, as for this poinsettia model system. This study is also unique in the sense that we have never seen a comparison between so distantly related species showing consistent results with respect to elucidating upon the genes involved in abscission, and at the same time comparing a system for primary abscission using mutants with an inducible system for secondary abscission. There is a large potential for further elucidation into the significance of the genes in this study, since poinsettia and pea are so far apart evolutionarily (Bennett et al., 2000). We have revealed at least 18 putative genes involved in the poinsettia secondary abscission process, many of which are expressed differentially also for primary abscission in peas. Earlier studies have tried to unravel the primary abscission process on a broader level using *Arabidopsis* as model (Wang et al., 2006), recently also González-Carranza et al. (2012).

Abscission is a complex process to unravel and we have compared poinsettia abscission with abscission-less (AZL) mutants first in *Arabidopsis*, with the interesting gene *inflorescence deficient in abscission* (*ida*) T-DNA mutant, identified as a novel putative ligand in plants (Butenko et al., 2003). The 35S:IDA line overexpressing *IDA* results in earlier abscission of floral organs and additionally abscission of organs that are not normally shed, like the whole silique. There is evidence that IDA encodes for arabinogalactan (Stenvik et al., 2006), a protein also found in naturally abscission surfaces. In our hands, poinsettia does not have any *ida* or *ida*-like genes. Although similar abscission processes take place in both species, the organs that abscise are not the same, since *Arabidopsis* sheds its petals, while poinsettia abscises the whole floral organ by developing an AZ on the pedicel. In *Arabidopsis* the pedicels do not abscise and there is only a rudiment of an AZ left at the base of the pedicles in this species, showing that AZ in *Arabidopsis* petals are primary (pre-defined). When we failed to find *ida* in poinsettia, we turned to the AZL *def* mutants in pea (Ayeh et al., 2011). The pea system demonstrated expression of highly conserved poinsettia sequences involved in pea abscission. Although poinsettia and *pea* are distantly related and the sequence homology is not expected to be high, we used our poinsettia *in situ* probes on pea *def* mutant tissue and obtained positive hybridization results (Ayeh, 2008).

### Gene sequences in common for pea and poinsettia during abscission

We will briefly discuss the genes with sequences common to poinsettia and pea abscission, i.e., common for primary and secondary abscission in our models. The potentially most important gene sequence could be 90a (XM_011094475/ AY188755). This riboprobe is only expressed in the wild type pea, and is upregulated during the first 2 days after poinsettia decapitation. This is a strong indicating that this gene is necessary for abscission to takes place, both in pea and poinsettia. Blast search revealed that 90a is close to an accession encoding an organ-specific protein (XM_011094475). Additional information, from the Blastx we did in 2012, gave a high similarity also to an atypical receptor-like kinase (AY188755). Since this is such a prominent gene sequence in our results, with a possibility that this may be one of the genes controlling abscission in plants, it would be very interesting to follow up on this.

*DD 38b* is a riboprobe expressed from the start of the abscission process and then downregulated from Day 3 in poinsettia. It is also expressed in the non-abscising mutants of pea. This suggests that this gene could be prohibiting abscission from taking place and the early expression pattern suggests a putative equally important, but opposite role as 90a in starting the cascade of events leading to abscission.

*DD 6a* (description Photosystem II subunit X) is found in both poinsettia and pea. Liao and Burns (2012) have found that Photosystem II subunit Q, X, P, and K had a two-fold increase in huanglongbing (HLB)-infected *Citrus sinencis* trees, where this infection was highly associated with both leaf and fruit abscission. They speculate whether this could be due to a breakdown of the photosynthetic apparatus since phloem plugging leads to starch accumulation in the leaves. In poinsettia, this gene is upregulated in Day 1 (after decapitation), before any visible signs of the AZ. In an AZ there is certainly a stop in the transport of photosynthetic products as the detachment is starting from the outside of the pedicel and moves inwards as the abscission process progresses during the week from decapitation to detachment, as can be seen in Figure 2, however, this is not happening until Days 4–6. Our results suggest that it is the gene for Photosystem II subunit X which is shutting down photosynthesis as an early event, and then there is a breakdown of the transport of sugars, causing the pedicel to start to yellow and senesce after another 2–4 days.

DD # 084 is annotated to Opie3 pol protein found in *Zea mays*. It is a receptor kinase, belonging to a large gene family in plants with more than 600 members in *Arabidopsis*. This gene family is said to be involved in a broad range of developmental processes, including abscission (Yu et al., 2003).

DD # 220 has a high similarity (81% and 2e-26) to a putative transcription factor from *R. communis*, also belonging to the *Euphorbiaceae* family, making this similarity even more likely. It has a strong upregulation in Day 2 and may be another putative key regulator. Transcription factors are often the key to the whole cascade of events for such complicated processes as abscission. As yet, we do not know which transcription factor it could be.

DD # 6a is expressed all through the abscission event, but is at its strongest upregulated on Day 1 of all the riboprobes, except # 82a, which also has its peak on Day 1. This is very early on, only 24 h after decapitation. It resembles a UVB-repressible protein from *Trifolium pretense*. This may have implications for the handling of stress in plants, as it is well-known that UVB induces stress and DNA damage in organisms.

DD # 25a is barely expressed on the day of decapitation, but expression increases strongly the day after. It resembles a gene from an unknown mitochondrial region in *Citrullus lanatus*. Very hard to say anything more on what this may imply, but the sequence being expressed in both poinsettia and wild type pea, suggests a closer look at the function.

### A proposed new phase division for secondary abscission

The pioneers of using the model system in *Arabidopsis* for studying abscission (Bleecker and Patterson, 1997), proposed to divide the abscission process into phases. They described this very well for primary abscission in *Arabidopsis*. However, we find that the development and morphology during induced secondary abscission in poinsettia needs to be divided with a higher resolution. We describe in a previous study the sequential transformations in the detection of cell wall polysaccharides during this induced abscission event (Lee et al., 2008) in the poinsettia model system. Based upon these results, as well as the present results on gene expression and the sequence of events here, we propose six phases: from Phase 0 to Phase V (Table 3). The induction phase (phase 0) is the induction and localization of the secondary abscission zone position, the following phase I is the where the cells become receptive to the signaling of the onset of the abscission process and where we observe the first biochemical changes (Lee et al., 2008). Phase II is characterized by cell divisions and the onset of rounded cells and continued biochemical changes, as can be seen by color changes in the distal part of the pedicel. Major biochemical restructuring characterize Phase III, where the ring marking the AZ is clearly visible and the analysis show de-esterification of homogalacturonan and the breakdown of cellulose, arabinose and other pectins preparing for the separation of the organ (Lee et al., 2008). Following the previously described breakdowns, the cell walls are enriched with xylan and lignin, probably to seal off the AZ once the distal part has been removed. We propose to call this Phase IV. Finally, Phase V represents the detachment of the organ. We have related the annotated gene sequences below to these proposed, more detailed phases of abscission.

### Annotated gene sequences upregulated at the onset (phase I)

The genes differentially regulated just after the time of decapitation are putatively the key regulators of abscission. Naturally, these are of utmost interest. Main indications from the gene expression analysis (DD + qRT-PCR + RNA *in situ*) gives the following zone specific regulations: The energy state is especially upregulated on Day 1 and 2, indicated by several hits on photosystem II [XP_007012396 and XM_002512633 (fk506-binding protein)] and mitochondrial genome (GQ856147 and HQ874649) related genes/proteins. Protein metabolism is specifically upregulated during Day 2 and 3, indicated by hits on a transcription factor (XM_002528833), transportRNA (AY794600), translation initiation (XM_002532624), and RNA binding protein (XM_002512412) related genes/proteins. There is initially (Days 0–2) an increase in cell divisions going on, but this is followed by downregulation as indicated by Histone deacetylase (XM_002511291) and α-tubulin (FJ228477). All these up-and downregulations are as expected from previous observations in the microscope on cells changing shape as the ordinary pedicel cells are initiated to form AZ cells, which are smaller, more dense and less vacuolated (Figure 3).

From Tables 1, 2, Day 2 is regulatory very important, where overall central proteins (strongly upregulated) can be the proteasome-like genes upregulated Day 2 (XM_012216503 and XM_002305663). We also find the V-ATPase G subunit 1 (XM_010526012) really strongly upregulated on Day 2. Burr et al. (2011) support that V-ATPase is associated with abscission in *Arabidopsis*.

Another strong upregulation is the Atypical receptor-like kinase from *Z. mays* (AY188755). Receptor-like kinases regulate a range of signaling pathways, many of which have been shown to be involved in abscission, as reviewed by Taylor and Whitelaw (2001). Since then, numerous groups have shown the importance of receptor-like kinases in abscission, examples can be: in *Arabidopsis* (Wagner and Kohorn, 2001; Butenko et al., 2003; Diévart and Clark, 2003; Cai and Lashbrook, 2008; Cho et al., 2008; Liljegren et al., 2009; González-Carranza et al., 2012) and in tomato (van der Hoorn et al., 2005).

We had another hit for an Organ-specific protein from *Sesamum indicum*. This up-regulation is extreme and it is bound to be of importance. This is one of the common genes for poinsettia and peas described earlier (90a) and hence extremely interesting.

A much better described hit is the Lysine-specific histone demethylase 1-1 (Table 2) from *Arabidopsis (*DD # 133a). This enzyme is closely associated with the gene Flowering Time Locus (FLC), which is in the core of events for *Arabidopsis* flowering in the complex pathway involving particularly temperature and day length. The enzyme is involved in H3K4 methylation of target genes, including FLC and FWA. We also had a predicted hit (*e* = 0.0) with *Prunus mume* with the same enzyme, confirming it.

### Annotated gene sequences downregulated at the onset (phase I)

The downregulated proteins can be equally important, such as Cytochrome f subunit (PetA; YP_002720125). Cytochrome F is a crucial component of the photosynthetic electron transport chain of higher plants. The subunit PetA is one of four major subunits. It seems like photosynthesis is restricted already after Day 0, the day of decapitation. This makes sense as the bud has started on its road to doom, and no longer needs to spend energy on photosynthesis. The yellowing of the pedicels become visible around Days 3–4 and could be due to the downregulation of this gene.

Another downregulated gene (DD #103) codes for CASP-like protein (XM_012213718). Roppolo et al. (2014) recently reported on *AtCASPL1D2*, a gene encoding a CASP-like protein expressed in the AZ of *Arabidopsis*. Another gene, *AtCASPL2A2* is reported to be expressed in the floral organ abscission zone (González-Carranza et al., 2012). Roppolo et al. (2014) states that: “*AtCASPL5C3* is expressed in the floral organ abscission zone as well, but its early expression in floral buds precedes the activation of the abscission zone and the expression of most of the genes known to be involved in floral organ shedding.” This supports our hit on XM_012213718 from *Jatropha curcas* (in the same family as poinsettia, *Euphorbiaceae*) as important for organ abscission and the sequence of events precedes the activation of the AZ. We deduce from this that CASP-like proteins may be important for the AZ to be able to be activated, a prerequisite for abscission, perhaps.

The combination of protein biosynthesis and degradation suggests a protein change context during the early steps of poinsettia pedicel leaf abscission. (Agustí et al., 2012) found expression in the lateral abscission zone for three putative E2 ligase proteins. Uridylate kinase (XM_002510884) is an enzyme in pyrimidin metabolism. The Unknown protein (CP011890) DD # 45c) has a very strong downregulation.

The last sequence downregulated is a riboprobe with resemblance to the V-SNARE proteins (DD # 108, from Table 2). These are proteins involved in membrane assembly and hence important in growth and development. Table 2 shows that this gene is downregulated just after Day 0, which is a remarkable quick response to decapitation.

### Other annotated gene sequences involved during later phases II-V

There is much evidence that glucanases are involved in the cell wall degradation necessary to break down the middle lamella and enable cell separation (Roberts et al., 2002). It is, therefore, hardly surprising to find Glucan endo-1,3-β -D-glucosidase and a Putative β-glucosidase (DD # 82a) popping up in the DD analysis. The putative Putative β-glucosidase (DD # 82a) is upregulated on Days 1–2, very early on, possibly to start the dehision of the cells. Then, there is a downregulation for the phases II-IV, before the final expression level increases. The final is possibly to degrade the walls in phase V prior to abscission. The other DD # 91b seems to have two peaks, one on Day 2 and another on Day 6, in a similar fashion.

The hits for genes associated with endoreduplication (DD # 136b—fk506-protein and DD #3a—α-tubulin) are of particular interest. Endoreduplication is the process of preparation for cell division with the division of nuclei, but without the follow-up of a cell division. The results are cells with multiple nuclei, which a larger than cells with only a single nuclei (Sugimoto-Shirasu and Roberts, 2003). We have seen the enlarged cells toward the end of the abscission process, very much in line with what Wong and Osborne reported for *Echallium elaterium* (Wong and Osborne, 1978). These enlarged cells probably aid the plant in pushing the unwanted organ off. These genes are higher in Days 0–2, then downregulated and end up being upregulated again in Day 7 and in the distal parts. We have run a cell sorting of poinsettia cells from the various days after decapitation in a flow cytometer and find that indeed, the cells have increasing number of nuclei as the days pass (data not shown).

DD # 208 with our only hit for a poinsettia sequence (*E. pulcherrima*, Table 1) is close to a gene sequence coding for chloroplast tRNA-Leu. This sequence codes for tRNA involved in Leucine assembly in the chloroplasts, thus another gene important for photosynthesis. # 208 has its peak expression on Days 2–3 and then downregulated toward the end of events on Day 6.

### Gene sequences strongly differentiated, with unknown function

The riboprobes # 304a, although with a length of 463 bp, has no significant matches in the databases as of end of August 2015. It is strongly upregulated on Day 2 and then downregulated again, indicating a putative gene in a signaling pathway. The riboprobes # 113, #125b, and # 125c are all upregulated on Day 2, albeit not as strongly as # 304. However, they are all very interesting as putative regulating genes as well. Riboprobe # 50a behaves differently from the other unknown sequences; it is the only one with no significant matches which is downregulated on Day 1, culminating at the lowest level on Day 2, only to be strongly upregulated again from Day 3 and onwards. This is probably a gene coding for an inhibitory gene product in abscission. Our group has also compared the results from immunolabeling poinsettia pedicels using antibodies (JIM5, JIM7, LM5, and LM6) to describe changes in arabinogalactan, galacturonan, and esterification of homogalacturonan (HG) in poinsettia. The earliest detected temporal change (Day 2) in poinsettia was a loss of LM5 [(1→4)-β-D-galactan] epitope in the distal region. On Day 5, the AZ lost the JIM5 (partially metyl-esterified/unesterified HG) in the distal part of the pedicel. The FT-IR analysis (Fourier-Transform Infra-Red microscopy) indicated that lignin and xylan were abundant in the AZ and that lower levels of cellulose, arabinose and pectins were present at Day 7 compared to the initiation phase I. The observations in poinsettia indicate that the induction of a secondary abscission event results in a temporal sequence of cell wall modifications involving the spatial regulatory loss, appearance and/or remodeling of distinct sets of cell wall epitopes. LM6 [(1→5)-α-L-arabinan] epitopes in the AZ cells disappeared at Day 7 (Lee et al., 2008). If we compare these findings with cell wall changes in *Def* wild type AZ using monoclonal antibodies LM5 and LM6; we observed changes in cell wall epitopes of the AZ from young to mature seeds. The apparent absence of (1→4)-β-D-galactan and (1→5)-α-L-arabinan epitopes in the AZ of mature *Def* wild type seeds may reveal the involvement of the action of hydroxyl ions (OH^−^) produced by peroxidases (Cosgrove, 2005) which is known to cleave wall polysaccharides. In *def* mutant pea seeds, the absence of an abscission event at the seed/funiculus junction may be due to a structural defect in forming the AZ rather than changes in the cell wall epitopes (Ayeh, 2008).

## Conclusions

The majority of the significant differentially expressed genes were upregulated during the first or second day after the induction of abscission. At this stage, it is not possible to see any changes in the anatomy of the pedicel or any color changes in poinsettia. Many of the genes found can be functionally classified to energy metabolism, cell growth and division. We have also identified two sequences similar to cell wall degrading enzymes (glucan endo-1,3-β-glucosidase and another putative β-glucosidase), as well as some sequences, which can be associated with endoreduplication (fk506-binding protein and α-tubulin), and a putative signal transducer. Our results are very much in alignment of the proposed model for abscission in Citrus (Agustí et al., 2012). The comparability with Citrus strongly supports the poinsettia model system to be suitable for further insight into gene regulations of the secondary abscission process. Based on our molecular results, it seems appropriate to modify the previous model of phases in abscission, by introducing a higher resolution for secondary abscission.

We have demonstrated that poinsettia and peas share at least six genes involved in abscission, two at the onset, two in phase II-III and the last two genes allocated to phase VI-V. It would be of great interest to use our two systems to find key regulatory gene(s) for abscission conserved throughout the plant kingdom. A universal abscission induction signal for AZ now seems more likely to find, since the six genes involved in abscission of both poinsettia and pea must be highly conserved during evolution. Further studies on our sequence data might reveal key regulatory genes, as well as more genes involved in the complicated abscission process for the plant kingdom.

## Author contributions

AKHE devised and participated in all aspects of the studies. AKHE and CAM coordinated the logistics of the DD study, while AKHE and YKL did the same for the RNA *in situ* hybridization experiments. CMM and CAM contributed to designing poinsettia experiments, growing of the plants, harvested materials and carried out the DD experiments. CMM did the validations through Real-Time qRT-PCR. KOA executed the pea experiments through growing, harvesting and preparing the pea material, as well as the RNA *in situ* in pea. PR executed the same in the poinsettia RNA *in situ* experiments. YKL performed the in structural analysis in the microscope together with KOA and PR and improved some of the *in situ* experiments through new hybridizations. TM performed the RACE experiments and the subsequent Real-Time qRT-PCR of the full-length sequences. AKHE, YKL, CAM, CMM, TM, KOA, and PR participated at various times in the data analysis. CAM, CMM, YKL, and AKHE participated in writing the article. All authors have read and approved the final manuscript.

### Conflict of interest statement

The authors declare that the research was conducted in the absence of any commercial or financial relationships that could be construed as a potential conflict of interest.

## Abbreviations

AZ: abscission zone
DD: differentially expressed genes.
