# Supplementary material for: Primary and Secondary Abscission in Pisum sativum and Euphorbia pulcherrima—How Do They Compare and How Do They Differ?
Source: Front Plant Sci. 2016 Jan 26;6:1204. doi: 10.3389/fpls.2015.01204 (PMC4726753; doi:10.3389/fpls.2015.01204)
Supplement: Supplementary file 1 [file Table1.docx]

Supplementary Material

**Primary and secondary abscission –**

how do they compare and how do they differ?

***Anne Kathrine Hvoslef-Eide^1*^, Cristel Munster^1^, Cecilie A. Mathiesen^1^, Kwadwo O. Ayeh^1,2^, Tone I. Melby^1^, Paoly Rasolomanana^1,3^ and YeonKyeong Lee^1^***

^1^Department of Plant Sciences, Norwegian University of Life Sciences, Aas, Norway.

^2^Present address: Department of Botany, School of Biological Sciences, College of Basic and Applied Sciences, University of Ghana, Legon-Accra, Ghana.

^3^Present address: Academic Program Directorate, Hawassa University, Ethiopia.

***Correspondence:** Anne Kathrine Hvoslef-Eide, Department of Plant Sciences, Norwegian University of Life Sciences, Box 5003, N-1432 Aas, Norway.

E-mail: [trine.hvoslef-eide@nmbu.no](mailto:trine.hvoslef-eide@nmbu.no)

**
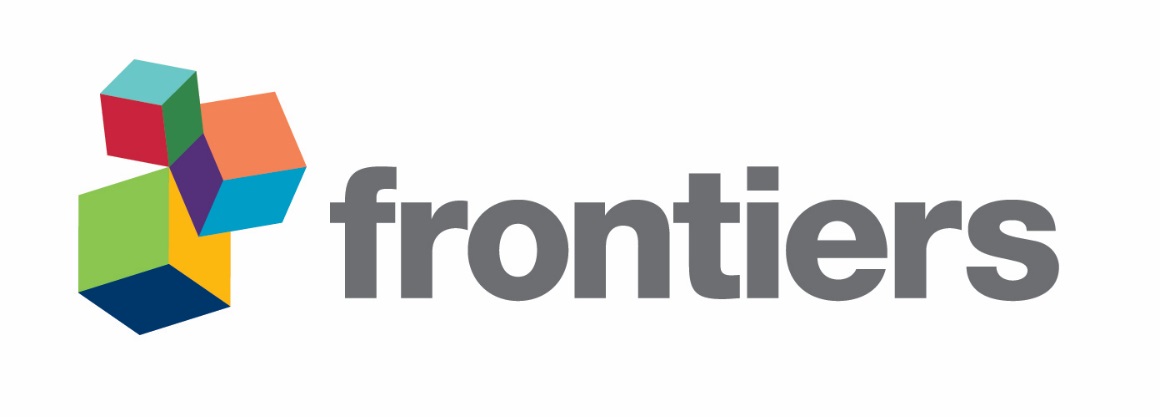
**

## Supplementary Tables

**Supplementary Table 1.**  **Real-Time RT-PCR primers for confirmation of DD expression of sequences isolated from the poinsettia flower abscission zone.**

| Clone | *Forward primer****^a^*** | *Reverse primer***^a^** |
| --- | --- | --- |

| 18S**^b^** | 5'-TGGTGCCCTTCCGTCAATT-3' | 5'-ACCATAAACGATGCCGACCA-3' |
| --- | --- | --- |
| 3a | 5'-TTATGAGGAGGTTGGTGCTGAG-3' | 5'-CTTTGAAGCAAAGAGAGCTCCA-3' |
| 6a | 5'-TTCCTCAAGTGGCTGAAGCTG-3' | 5'-GGTCGAAATTGGAGACACCAAT-3' |
| 25a | 5'-AGCTGCTCTTTGAAGTTCCTCG-3' | 5'-AACCGTTTATGCGGCCACT-3' |
| 32 | 5'-ATCAACCCCCAAAGGAGAAA-3' | 5'-TGAAGGTAGAGGCAGGCTTT-3' |
| 38b | 5'-TGGACTGTCTGTTTTAGTGTGTCAA-3' | 5'-AGCCTCAAAAAACACTTCTCCAG-3' |
| 45c | 5'-CCGTAACGAGGATCGAGTTGAT-3' | 5'-CATTGTCTTGCGTAGTTTTCCTTTC-3' |
| 47b | 5'-GGTGACGCCATGGAAGAATG-3' | 5'-TGTGCGGAAAATCGGAAGTC-3' |
| 50a | 5'-ATGCCACCAATCCATGCCTA-3' | 5'-TAACCCCATTCCGCTGCTACT-3' |
| 57b | 5'-TACCTCCAGGACCGGAACTTCT-3' | 5'-TATTTCCGCATCCCCCTGA-3' |
| 60c | 5'-GAGGCCTCGAGAATTTCCCAT-3' | 5'-CCGAACGCCGATATTCAAATC-3' |
| 82a | 5'-CCCTTGTCTCTGGCTCACTTCTT-3' | 5'-GGCAGAATCAAAAGCATCCAAA-3' |
| 82b | GGTGATGTCACCAGTGCAAG-3' | 5'-CACGAATATTTCGCCCTTGT-3' |
| 84_ | 5'-GTGTGCAAAATCCAGTGCTGAC-3' | 5'-CACCCAAATCCCAAACTTCG-3' |
| 90a | 5'-AGCCCATATATTCCTAATCTTTCGG-3' | 5'-CATCGTACACCACCGGATCA-3' |
| 91b | 5'-GCCATGGCTCCTTTTTTAGC-3' | 5'-CGAGAGGATCCATAATTACAAATCG-3' |
| 101_ | 5'-TGACGAGGAAAAGAAGCCCA-3' | 5'-CGCTCCCTCCTCTCAACTTTTA-3' |
| 103_ | 5'-GAGTAACCTGAACCGCACTGGA-3' | 5'-TCAACAGCCAAAGCAGCCA-3' |
| 105 | 5'-ATGTGATTCAATGCCGTGAG-3' | 5'-GATATGACTTTCTTTGATTCCATGC-3' |
| 113_ | 5'-TCCTTAACCCAGTTGTTGGCC-3' | 5'-CCCCTTTCTGATTGCAGCAA-3' |
| 122 | 5'-GTGCAGCAAAAACGCTAACA-3' | 5'-GCAGGGTGTCAACACTCAAG |
| 125b | 5'-TGCGACTTGTGTTGTGAGGATC-3' | 5'-CCACGATGTAACACCCCCTTAA-3' |
| 125c | 5'-GACCAGCTATTCGTGTCTTGGC-3' | 5'-CCCATGTCAACAATGCAGACAA |
| 130b | 5'-GTTGGTGCGGCTGAATTTT-3' | 5'-ATAGTGGTTAACTGATGATGCAGCT-3' |
| 133a | 5'-GGCGGTGACCATTGTTTTAT-3' | 5'-TCACCTCGAACCTCTTGCTT-3' |
| 135 | 5'-TTGCATGACACCTTCTCTGC-3' | 5'-TGAGCAGCATACCATTCAGC-3' |
| 136b | 5'-AATGCAGGATAGTTGTGCCACC-3' | 5'-AAAATCTAAGGCCCTCTGCCC-3' |
| 140a | 5'-TTATGGCATGGCTTTAGGCG-3' | 5'-CCTGGTTCCTCAGCACAAAATC-3' |
| 140b | 5'-TCCAACCCAATGTGTACGAGC-3' | 5'-TTCATCAGGCAGTTTGCGC-3' |
| 204c | 5'-TGCTGTTGCAGAGGAAGGTTC-3' | 5'-CCCATGTCTTTACTGCCATTCA-3' |
| 208_ | 5'-AAAACGAGGAAGACAGCGCA-3' | 5'-TGCCAGGAACCAGATTTGAACT-3' |
| 220_ | 5'-GCTGCTTGTTGTGCTGCAATT-3' | 5'-AAAGGTCAGAGCTTTTCCCCG-3' |
| 301_ | 5'-GAATCCGACGAGGATGAGGAA-3' | 5'-TCCCCAAACTCCGACCAAA-3' |
| 304a | 5'-GCCATGTTGCTGTAACTGAAGC-3' | 5'-TGAGACTCTTGTGCTCCGAGTG-3' |
| 304b | 5'-TTGGATCCTAGAGAGGCGGAA-3' | 5'-TGATCAGAAGCATCCAGCAGC-3' |
| 320b | 5'-GATCCGGTGGTGTACGATGA-3' | 5'-CAGACAGCTAGTAAAGGACTTGCAG-3' |

**^a^** Primers were synthesized by Invitrogen

**^b^** 18S was used as normalization reference
