# Supplementary material for: Primary and Secondary Abscission in Pisum sativum and Euphorbia pulcherrima—How Do They Compare and How Do They Differ?
Source: Front Plant Sci. 2016 Jan 26;6:1204. doi: 10.3389/fpls.2015.01204 (PMC4726753; doi:10.3389/fpls.2015.01204)
Supplement: Supplementary file 2 [file Table2.docx]

Supplementary Material

**Primary and secondary abscission –**

how do they compare and how do they differ?

***Anne Kathrine Hvoslef-Eide^1*^, Cristel Munster^1^, Cecilie A. Mathiesen^1^, Kwadwo O. Ayeh^1,2^, Tone I. Melby^1^, Paoly Rasolomanana^1,3^ and YeonKyeong Lee^1^***

^1^Department of Plant Sciences, Norwegian University of Life Sciences, Aas, Norway.

^2^Present address: Department of Botany, School of Biological Sciences, College of Basic and Applied Sciences, University of Ghana, Legon-Accra, Ghana.

^3^Present address: Academic Program Directorate, Hawassa University, Ethiopia.

***Correspondence:** Anne Kathrine Hvoslef-Eide, Department of Plant Sciences, Norwegian University of Life Sciences, Box 5003, N-1432 Aas, Norway.

E-mail: [trine.hvoslef-eide@nmbu.no](mailto:trine.hvoslef-eide@nmbu.no)

**
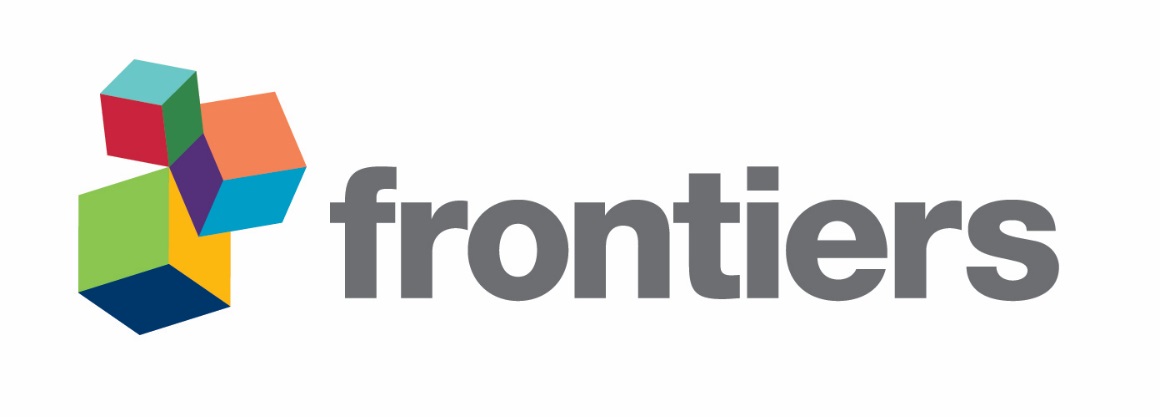
**

## Supplementary Tables

**Supplementary Table 2. Oligonucleotides used in 5`RACE for poinsettia DD sequences**

| **BD SMART II A Olgonucleotide** | **5`- AAGCAGTGGTATCAACGCAGAGTACGCGGG-3`** |
| --- | --- |
| **5`-RACE CDS Primer A** | **5`- (T)_25_VN-3` (N=A,C,G, or T ; V=A,G, or C)** |
| **Long Universal Primer** | **5`- CTAATACGACYCACTATAGGGCAAGCAGTGGTATCAACGCAGAGT-3`** |
| **Short Universal Primer** | **5`- CTAATACGACTCACTATAGGGC-3`** |
