# Supplementary material for: Primary and Secondary Abscission in Pisum sativum and Euphorbia pulcherrima—How Do They Compare and How Do They Differ?
Source: Front Plant Sci. 2016 Jan 26;6:1204. doi: 10.3389/fpls.2015.01204 (PMC4726753; doi:10.3389/fpls.2015.01204)
Supplement: Supplementary file 3 [file Table3.docx]

Supplementary Material

**Primary and secondary abscission –**

how do they compare and how do they differ?

***Anne Kathrine Hvoslef-Eide^1*^, Cristel Munster^1^, Cecilie A. Mathiesen^1^, Kwadwo O. Ayeh^1,2^, Tone I. Melby^1^, Paoly Rasolomanana^1,3^ and YeonKyeong Lee^1^***

^1^Department of Plant Sciences, Norwegian University of Life Sciences, Aas, Norway.

^2^Present address: Department of Botany, School of Biological Sciences, College of Basic and Applied Sciences, University of Ghana, Legon-Accra, Ghana.

^3^Present address: Academic Program Directorate, Hawassa University, Ethiopia.

***Correspondence:** Anne Kathrine Hvoslef-Eide, Department of Plant Sciences, Norwegian University of Life Sciences, Box 5003, N-1432 Aas, Norway.

E-mail: [trine.hvoslef-eide@nmbu.no](mailto:trine.hvoslef-eide@nmbu.no)

**
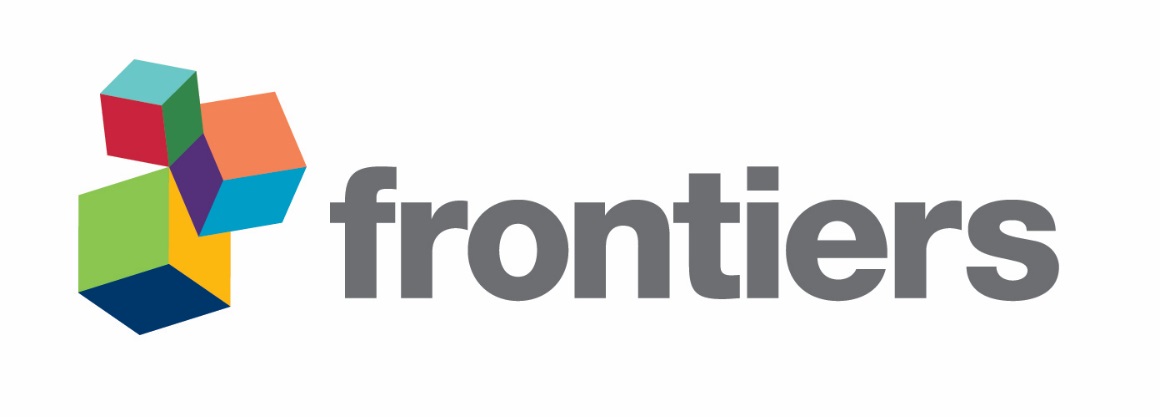
**

## Supplementary Tables

**Supplementary Table 3**. **Primer-pairs for the seven full-length genes from poinsettia to q-PCR reaction with Tm, length (nt) and product size respectively, together with 18S and α –tubulin for comparison.**

| **Gene** | **5`-3`Left primer** | **Tm** | **mer** | **5`-3`Right primer** | **Tm** | **nt** | **Product size** | **Location on sequence** |
| --- | --- | --- | --- | --- | --- | --- | --- | --- |
| 18s | aaacggctaccacatccaag | 59.99 | 20 | cctccaatggatcctcgtta | 59.89 | 20 | 153 bp |  |
| α -tubulin | gaggaggttggtgctgagtc | 59.84 | 20 | tgaagcaaagagagctccaaa | 60.26 | 21 | 150 bp |  |
| D0-135 | ttgcatgacaccttctctgc | 59.99 | 20 | tgagcagcataccattcagc | 59.98 | 20 | 151 bp | 749 - 899 bp |
| D0-105 | atgtgattcaatgccgtg ag | 59.53 | 20 | gatatgactttctttgattccatgc | 60.22 | 25 | 140 bp | 189 – 328 bp |
| D0-108a | gggcaagcagtggtatcaac | 60.53 | 20 | cgatctggtatgatcggaca | 59.48 | 20 | 153 bp | 19 – 171 bp |
| D2-82b | ggtgatgtcaccagtgcaag | 60.16 | 20 | cacgaatatttcgcccttgt | 59.96 | 20 | 148 bp | 281 – 428 bp |
| D2-122 | gtgcagcaaaaacgctaaca | 60.06 | 20 | gcagggtgtcaacactcaag | 59.31 | 20 | 156 bp | 507 - 664 bp |
| D2-133a | ggcggtgaccattgttttat | 59.69 | 20 | tcacctcgaacctcttgctt | 59.99 | 20 | 155 bp | 1342 – 1496 bp |
| D4-32 | atcaacccccaaaggagaaa | 60.66 | 20 | tgaaggtagaggcaggcttt | 59.08 | 20 | 150 bp | 226 – 375 bp |
