# Supplementary material for: Primary and Secondary Abscission in Pisum sativum and Euphorbia pulcherrima—How Do They Compare and How Do They Differ?
Source: Front Plant Sci. 2016 Jan 26;6:1204. doi: 10.3389/fpls.2015.01204 (PMC4726753; doi:10.3389/fpls.2015.01204)
Supplement: Supplementary file 4 [file Table4.docx]

Supplementary Material

**Primary and secondary abscission –**

how do they compare and how do they differ?

***Anne Kathrine Hvoslef-Eide^1*^, Cristel Munster^1^, Cecilie A. Mathiesen^1^, Kwadwo O. Ayeh^1,2^, Tone I. Melby^1^, Paoly Rasolomanana^1,3^ and YeonKyeong Lee^1^***

^1^Department of Plant Sciences, Norwegian University of Life Sciences, Aas, Norway.

^2^Present address: Department of Botany, School of Biological Sciences, College of Basic and Applied Sciences, University of Ghana, Legon-Accra, Ghana.

^3^Present address: Academic Program Directorate, Hawassa University, Ethiopia.

***Correspondence:** Anne Kathrine Hvoslef-Eide, Department of Plant Sciences, Norwegian University of Life Sciences, Box 5003, N-1432 Aas, Norway.

E-mail: [trine.hvoslef-eide@nmbu.no](mailto:trine.hvoslef-eide@nmbu.no)

**
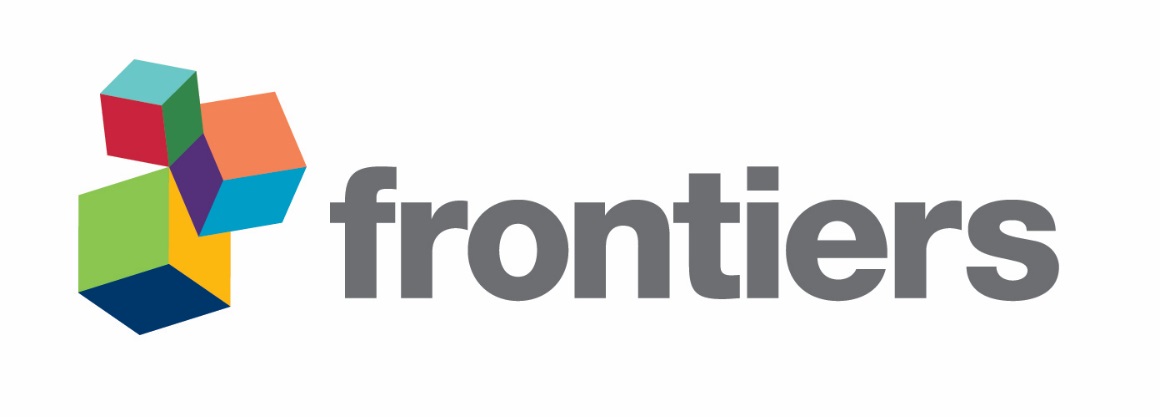
**

## Supplementary Tables

**Table 4*.* *In situ hybridization* primers for confirmation of DD expression of sequences isolated from the poinsettia flower abscission zone. Probes were used for RNA *in situ* in pea.**

| Clone | *Forward primer^a^* | Reverse primer^a^ |
| --- | --- | --- |
| 6a | 5'-TTCCTCAAGTGGCTGAAGCTG-3' | 5'-GGTCGAAATTGGAGACACCAAT-3' |
| 25a | 5'-AGCTGCTCTTTGAAGTTCCTCG-3' | 5'-AACCGTTTATGCGGCCACT-3' |
| 38b | 5'-TGGACTGTCTGTTTTAGTGTGTCAA-3' | 5'-AGCCTCAAAAAACACTTCTCCAG-3' |
| 84_ | 5'-GTGTGCAAAATCCAGTGCTGAC-3' | 5'-CACCCAAATCCCAAACTTCG-3' |
| 90a | 5'-AGCCCATATATTCCTAATCTTTCGG-3' | 5'-CATCGTACACCACCGGATCA-3' |
| 220_ | 5'-GCTGCTTGTTGTGCTGCAATT-3' | 5'-AAAGGTCAGAGCTTTTCCCCG-3' |

^a^ Primers were synthesized by Invitrogen

^b^ 18S was used as normalisation reference
